# Comprehensive molecular analyses of a circadian rhythm-related gene diagnostic model and immune infiltration using machine learning methods in sepsis-associated acute kidney injury

**DOI:** 10.1186/s41065-026-00698-3

**Published:** 2026-06-11

**Authors:** Yuzhen Luo, Tangjuan Liu, Yiqiang Chen

**Affiliations:** 1 Department of Nephrology, First Affiliated Hospital of Guangxi Medical University, Nanning, 530021 China; 2 Department of Pulmonary and Critical Care Medicine, First Affiliated Hospital of Guangxi Medical University, Nanning, 530021 China

**Keywords:** Sepsis-associated acute kidney injury, Circadian rhythm, Biomarker, Machine learning, Lasso, SVM-RFE, Random forest

## Abstract

**Background:**

This study aimed to explore hub circadian rhythm-related genes (CRRGs) associated with sepsis-associated acute kidney injury (saAKI) using machine learning algorithms to provide novel ideas for the diagnosis and treatment of this disease.

**Materials:**

Two AKI datasets (GSE30718 and GSE139061) were obtained from the Gene Expression Omnibus (GEO) database. We initially identified differentially expressed genes (DEGs) and performed WGCNA to identify candidate CRRGs. Following this, diagnostic CRRGs were identified on the basis of machine learning algorithms. The ssGSEA method was used to measure the link between hub CRRGs and immune cells. GSEA and GSVA were used to understand the relevant functions and pathways. The hub CRRGs were validated using the GSE255281 dataset and RT‒qPCR of clinical blood samples.

**Results:**

On the basis of the DEGs and WGCNA results, a total of 21 candidate CRRGs were obtained for saAKI. Seven diagnostic CRRGs were identified via the Lasso, random forest (RF) and SVM-RFE algorithms. We then constructed a predictive nomogram using the diagnostic CRRGs, and the calibration, decision and ROC curves revealed that it had high diagnostic efficiency. Through the evaluation of 2 subdatasets, 4 hub CRRGs (DEFB1, EGF, REN and PTPRD) were ultimately identified. Immune cell infiltration analysis indicated that there might be immune dysregulation in saAKI. GSEA and GSVA revealed different biological functions and pathways related to immunity, inflammation, metabolism and material transport that might exist in different subgroups of hub CRRGs. The validation dataset GSE255281 and RT‒qPCR analysis verified the expression of EGF and PTPRD.

**Conclusion:**

Our research utilized comprehensive machine learning methods to construct a CRRG-based diagnostic model for saAKI. The identified CRRGs may be potential biomarkers of saAKI, and this study may provide new strategies for the diagnosis and treatment of saAKI.

**Supplementary Information:**

The online version contains supplementary material available at 10.1186/s41065-026-00698-3.

## Introduction

Acute kidney injury (AKI) is among the most common complications of sepsis, with a high incidence of 47–61% [1]. Patients with sepsis complicated with AKI have poor prognoses and significantly higher mortality [2]. In addition, patients with sepsis-associated AKI (saAKI) are associated with an increased risk of long-term chronic kidney disease [3]. Therefore, prevention, early diagnosis and treatment are vital for this disease. At present, several novel indicators, such as neutrophil gelatinase-associated lipocalin (NGAL) and kidney injury molecule-1 (Kim-1), can be used to detect AKI; however, these methods have many limitations, such as low specificity under specific conditions, a lack of test availability and cost-effectiveness issues [4]. Thus, it is necessary to explore the specific characteristics of saAKI, which will also help to elucidate its biological mechanisms.

The circadian rhythm is a manifestation of the human body clock and plays important roles in many physiological processes, including metabolism, immunity and cell death, which coordinate the behavior and physiology of all organs to achieve system homeostasis [5, 6]. The circadian rhythm can regulate immune and inflammatory responses, and the circadian/metabolic axis may be closely related to the rhythm of immune function [7]. Disturbance of the circadian rhythm can cause dysfunction of the body, including abnormal cell proliferation, increased mutation, and apoptosis resistance, which can lead to the occurrence of many diseases [5, 8]. Previous studies have shown that circadian rhythms play a role in the onset and development of AKI [9, 10]. Shi et al. found that different times of day have different effects on cholesterol crystal embolism-related AKI, which is related to the circadian rhythm of neutrophil recruitment [9]. The renin-angiotensin-aldosterone system (RAAS) has a circadian rhythm, and early regulation of the circadian rhythm of key components of the RAAS can prevent or reduce the severity of AKI that may occur with COVID-19 [10]. Recent studies suggest that circadian rhythm-related genes (CRRGs) can be used as biomarkers for the diagnosis, prognosis and immunology of diseases [11–13]. However, the current research on the effects of circadian rhythm on AKI patients is still limited, and systematically exploring the characteristics of CRRGs may provide new methods for the diagnosis and treatment of AKI.

The rapid development of high-throughput transcriptomic techniques and bioinformatics enables researchers to investigate genetic changes in various diseases and identify biomarkers for diagnosis and treatment. Machine learning is a new type of algorithm and statistical model that has been applied by an increasing number of researchers to screen and identify disease biomarkers [14, 15]. Least absolute shrinkage and selector operation (Lasso) and support vector machine recursive feature elimination (SVM-RFE) are used to reduce overfitting when screening important variables [16, 17]. Algorithm weighted gene coexpression network analysis (WGCNA) clusters genes into different modules, reveals the relationships between modules and disease traits, and identifies the core genes in the network [18]. Integrated machine learning algorithms have proven to be effective tools for identifying hub genes of disease [11, 12, 16, 19–21]. Thus, we synthesized the evidence from multiple machine learning algorithms to identify more reliable molecular markers in saAKI.

This study systematically explored the CRRGs and molecular mechanisms closely linked to saAKI. First, differentially expressed gene (DEG) analysis was combined with WGCNA to identify candidate genes on the basis of clinical characteristics. The key CRRGs in saAKI were then identified on the basis of the candidate genes using the Lasso, SVM-RFE and RF algorithms. On the basis of key CRRGs, we constructed a diagnostic nomogram model for saAKI. Finally, we explored the potential molecular mechanisms of CRRGs in saAKI.

## Materials and methods

### Data sources and processing

The GEO database includes high-throughput gene expression data submitted by researchers around the world. In this study, 3 AKI mRNA datasets (GSE30718, GSE139061 and GSE255281) were selected for analysis. The GSE139061 dataset includes AKI patients diagnosed with sepsis, while the GSE30718 dataset consists of AKI patients after kidney transplantation. The GSE30718 dataset contains 28 AKI samples and 19 control samples, and the GSE139061 dataset contains 39 saAKI samples and 9 control samples. The GSE255281 dataset contains 10 mouse saAKI samples and 10 control samples. A total of 1,424 circadian rhythm-related genes (CRRGs) were obtained from a previous article [22]. First, we normalized the 2 datasets to remove batch effects and then merged them, which was conducted through the “limma” and “sva” packages. Principal component analysis (PCA) was used to determine the distribution of the samples from the saAKI and normal groups and to assess the situation before and after batch effect removal. The GSE255281 dataset was used as a validation cohort.

### Identification of DEGs and weighted gene coexpression network analysis

We identified DEGs between the saAKI and control samples using the “limma” package. |LogFC| > 0.5 and adjusted *p* < 0.05 were used as screening criteria for DEGs. A volcano map was constructed to show the significantly differentially expressed genes. To comprehensively investigate the genetic mechanism of AKI pathogenesis, we constructed a coexpression network using the “WGCNA” package [18]. We selected genes whose median absolute deviation (MAD) was > 25% for subsequent analysis. Next, the “goodSamplesGenes” and “pickSoftThreshold” functions were used to determine and verify the optimal soft threshold (b), and a coexpression network was constructed. We then used the hierarchical clustering method to construct a dendrogram and assess the relationship between the module feature genes and the disease. The modules that were strongly correlated with saAKI were ultimately selected, and the genes in the module were used for subsequent analysis. The intersection genes of the module genes, DEGs and CRRGs were selected as candidate genes.

### Correlation analysis and functional enrichment analysis

Afterward, we comprehensively analyzed the candidate CRRGs. The chromosomal localization of these candidate CRRGs was determined by the “RCircos” package. Pearson correlation analysis was performed to determine the expression correlations among these CRRGs. A protein‒protein interaction (PPI) network was constructed through the GeneMANIA website (https://genemania.org/) to further evaluate the interactions between candidate CRRGs. To explore the functions and pathways associated with saAKI, the “clusterProfiler” and “enrichplot” packages were used to perform GO and KEGG analyses. *P* < 0.05 was used as the significance threshold for the GO and KEGG results.

### Identification of diagnostic markers for saAKI

The Lasso, SVM-RFE and RF algorithms are typically used to screen genes and help reduce overfitting, as has been well illustrated by previous studies [23, 24]. On the basis of the above candidate CRRGs, we performed these 3 algorithms. The Lasso algorithm was executed using the “glmnet” package, with family= “binomial” set to apply to the candidate CRRG expression data. The key parameter alpha was set to 1, and a 10-fold cross-verification was performed. Finally, lambda.min was used as the lambda value to reduce the error of cross-verification. The SVM-RFE algorithm is executed using the “e1071” R package and is often used to screen model variables [25]. In this study, the parameters were set as follows: a 10-fold cross-verification (k = 10) was conducted on the expression data of candidate CRRGs; feature selection was performed through the svmRFE function with the parameter halve.above = 50; and features from 1 to 20 were measured by the FeatSweep.wrap function. The RF algorithm is carried out using the “randomForest” package. The importance of features was determined by the mean decrease Gini index, and the top 10 CRRGs in terms of importance were selected. The intersection of the 3 results was identified as the diagnostic CRRGs for saAKI.

### Construction and assessment of a diagnostic nomogram

On the basis of the diagnostic CRRGs, we constructed a diagnostic model using the “rms” package. Calibration curves, decision curve analysis, and ROC curves were used to evaluate the predictive performance of the nomogram model. The GSE255281 dataset was used as an external validation cohort to evaluate the accuracy of the model. To further evaluate the diagnostic efficacy of the selected CRRGs, we subsequently compared the diagnostic CRRG expression levels in normal and saAKI samples in the 2 datasets (GSE30718 and GSE139061) and used ROC curves to measure the diagnostic efficacy. Finally, the CRRGs with significant differences and the same trend were selected as the hub CRRGs.

### Assessment of immune cell infiltration

Immune cell infiltration levels in saAKI were assessed using the ssGSEA method via the “gsva” package [26]. We compared the levels of immune cells in the saAKI and control samples and assessed the overall difference between the two groups using PCA. In addition, we analyzed the relationship between the expression of the hub CRRGs and the level of immune cell infiltration by Spearman correlation analysis.

### GSEA and GSVA

The samples were divided into high- and low-expression groups according to the median expression value of each hub CRRG, and the potential biological functions related to each CRRG were evaluated by GSEA, while GSVA was used to explore the potential biological pathways. The HPA database (https://www.proteinatlas.org/) was utilized to further evaluate the expression of hub CRRGs in different cell subtypes at the single-cell level. The association of these hub CRRGs with AKI was evaluated through the Comparative Toxicogenomics Database (CTD) (http://ctdbase.org/).

### RT‒qPCR

To test the gene expression levels of DEFB1, EGF, REN and PTPRD in patients with saAKI and controls, quantitative real-time polymerase chain reaction (RT–qPCR) analysis of the mRNA levels of the genes from the whole blood of patients was performed (Solarbio, China). The concentration of total RNA was detected using an ultramicro nucleic acid analyzer with a sample volume of 1 µL. Qualified RNA samples have A260/A280 ratios ranging from 1.7 to 2.1; RNA samples that fail to meet this purity requirement require re-extraction. The mRNA was reverse transcribed to cDNA using an Omniscript RT kit (Yeasen, China). RT‒qPCR analysis was performed using Hieff^®^ qPCR SYBR Green Master Mix (Yeasen, China). The reaction conditions for PCR were as follows: pretreatment at 95 °C for 60 s; 35 cycles of denaturation at 95 °C for 5 s; annealing at 60 °C for 30 s; and extension at 72 °C for 30 s. The default standard melting curve program of the instrument was used. After ACTB normalization, the relative expression levels of the target genes were determined with the 2^-△△ct^ approach. The primer sequences are listed in Supplementary Table (1) A total of 20 patients who were diagnosed with saAKI at the First Affiliated Hospital of Guangxi Medical University in 2025 were included in the observation group, and 20 patients were included in the control group. The clinical characteristics of the SaAKI patients are shown in Supplementary Table (2) Whole blood samples were collected within 24 h after the confirmation of a diagnosis of saAKI. The inclusion criteria were as follows: ① met the Sepsis 3.0 diagnostic criteria [27], which require confirmed or suspected infection along with a Sequential Organ Failure Assessment (SOFA) score of ≥ 2 or a quick SOFA (qSOFA) score of ≥ 2; and ② met the diagnostic criteria for acute kidney injury [3, 28]. The exclusion criteria were as follows: ① patients with a history of prior renal surgery; ② patients receiving hemodialysis for acute or chronic kidney injury; and ③ patients with hematological or immunological diseases.

### Statistical analysis

In this study, the differences between groups were analyzed by the Wilcoxon test. Correlations among CRRGs were evaluated by Pearson analysis, while correlations between CRRGs and immune cell infiltration were evaluated by Spearman analysis. To analyze the RT‒qPCR results, an independent sample t test was used. In our research, R (v 4.1.2) was used for graphic production and analysis. *P* < 0.05 was considered to indicate statistical significance.

## Results

### Identification of DEGs and WGCNA

**Fig. 1:**
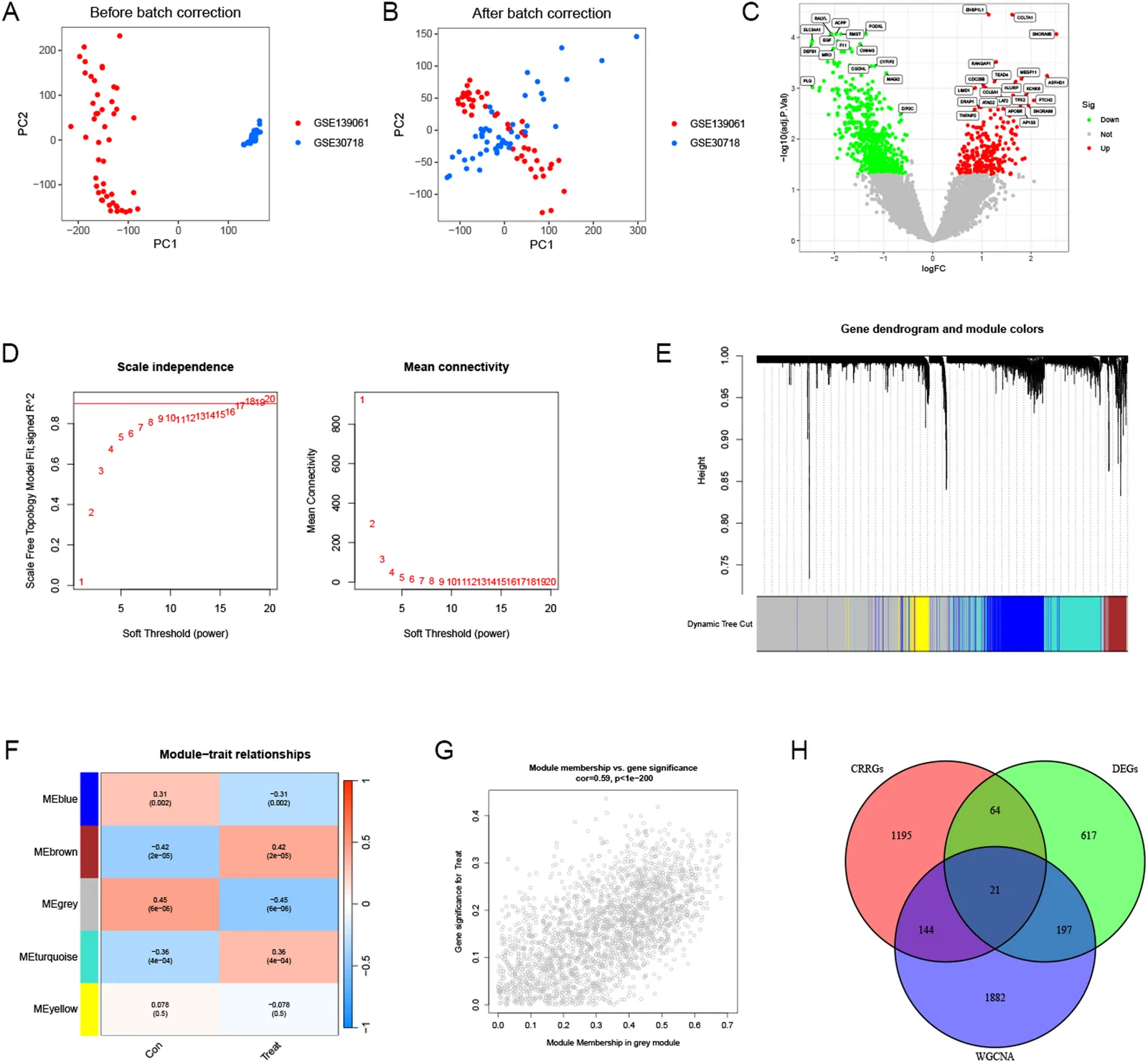
Candidate CRRGs were identified on the basis of the differentially expressed genes (DEGs) and WGCNA. (**A**–**B**) Principal component analysis shows the sample distribution before and after the 2 datasets were merged, revealing that the difference in the data between the 2 datasets after merging was relatively small. (**C**) DEGs between saAKI and normal samples are shown in a volcano map. (**D**) Scale-free fit index and network connectivity under different soft thresholds. (**E**) Clustering dendrogram and merging of the gene coexpression modules shown by different colors in saAKI. (**F**) The heatmap shows the relationships between the modules and clinical features, and the gray module genes are most strongly correlated. (**G**) The relationship between the module membership and gene significance in the gray module is shown. (**H**) The overlapping genes were used as candidate CRRGs

The 2 GEO datasets contain a total of 95 samples, and batch effects are removed through the “ComBat” feature of the “sva” package. PCA revealed the sample distribution before and after the merger, and the results revealed that the batch effect was effectively removed (Fig. 1A–B). A total of 898 DEGs were identified, and we constructed a volcano map of the DEGs between normal and saAKI samples (Fig. 1C). The WGCNA algorithm was used to determine the gene–gene modules associated with saAKI (Fig. 1D–E). Trait correlation analysis revealed that several modules were associated with saAKI, among which the gray module contained 2244 key genes and was most strongly correlated with both the positive saAKI group and the control group (Fig. 1F–G). Among the gray module genes, DEGs and CRRGs, a total of 21 shared genes were selected as candidate CRRGs (Fig. 1H).

### Expression and functional enrichment analysis of CRRGs

**Fig. 2:**
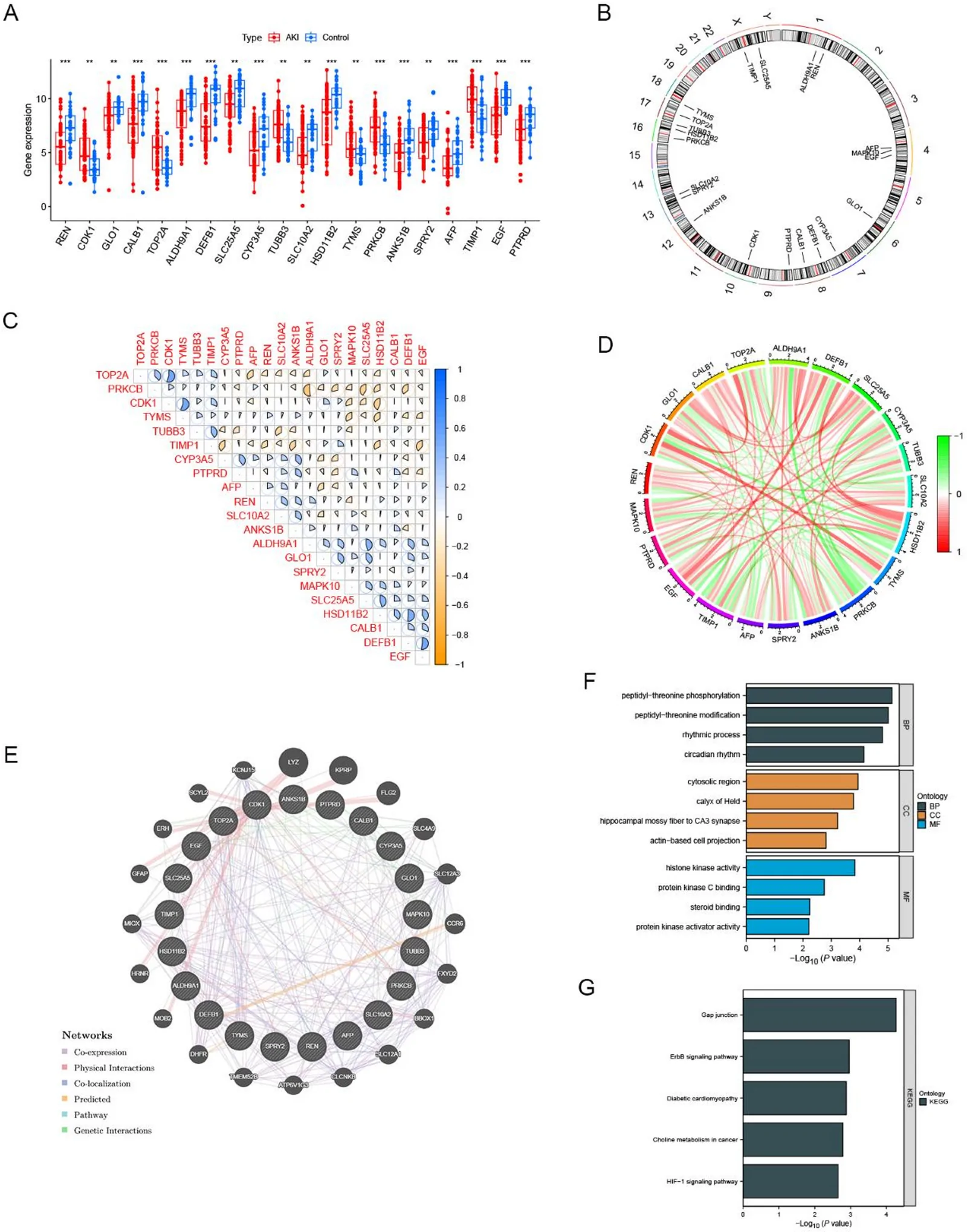
Functional enrichment analysis of candidate CRRGs in saAKI. (**A**) The results of the Wilcoxon test revealed that 20 out of the 21 CRRGs were dysregulated in saAKI. (**B**) Chromosome distribution of the candidate CRRGs. (**C**-**D**) Correlations among the expression of 21 CRRGs in saAKI. Blue and red represent positive correlations, whereas yellow and green represent negative correlations. (**E**) The PPI network generated in the MANIA database; the colors represent the potential correlations. (**F**) Results of the GO analysis of the candidate CRRGs. (**G**) KEGG analysis results for the candidate CRRGs. **P < 0.01, ***P < 0.001

Among the 21 candidate CRRGs, compared with those in the control samples, the expression of 14 CRRGs decreased and that of 6 CRRGs increased in the saAKI samples (Fig. 2A). We utilized a Circos track plot to show the chromosomal localization of these CRRGs (Fig. 2B). The correlation between the expression of the candidate CRRGs in saAKI is shown in Fig. 2C–D. The PPI network generated in the MANIA database shows the potential interactions of these CRRGs (Fig. 2E). GO analysis revealed that the 21 CRRGs might be involved in the circadian rhythm, rhythmic process, peptidyl−threonine modification, and peptidyl−threonine phosphorylation (BP); actin-based cell projection, hippocampal mossy fibers to CA3 synapses, calyx of Held, and the cytosolic region (CC); and protein kinase activator activity, steroid binding, protein kinase C binding, and histone kinase activity (MF) (Fig. 2F). KEGG pathway analysis revealed that the candidate CRRGs might be linked to the HIF-1 signaling pathway, choline metabolism in cancer, diabetic cardiomyopathy, the ErbB signaling pathway, and gap junctions (Fig. 2G).

### Identification of diagnostic CRRGs in saAKI

**Fig. 3:**
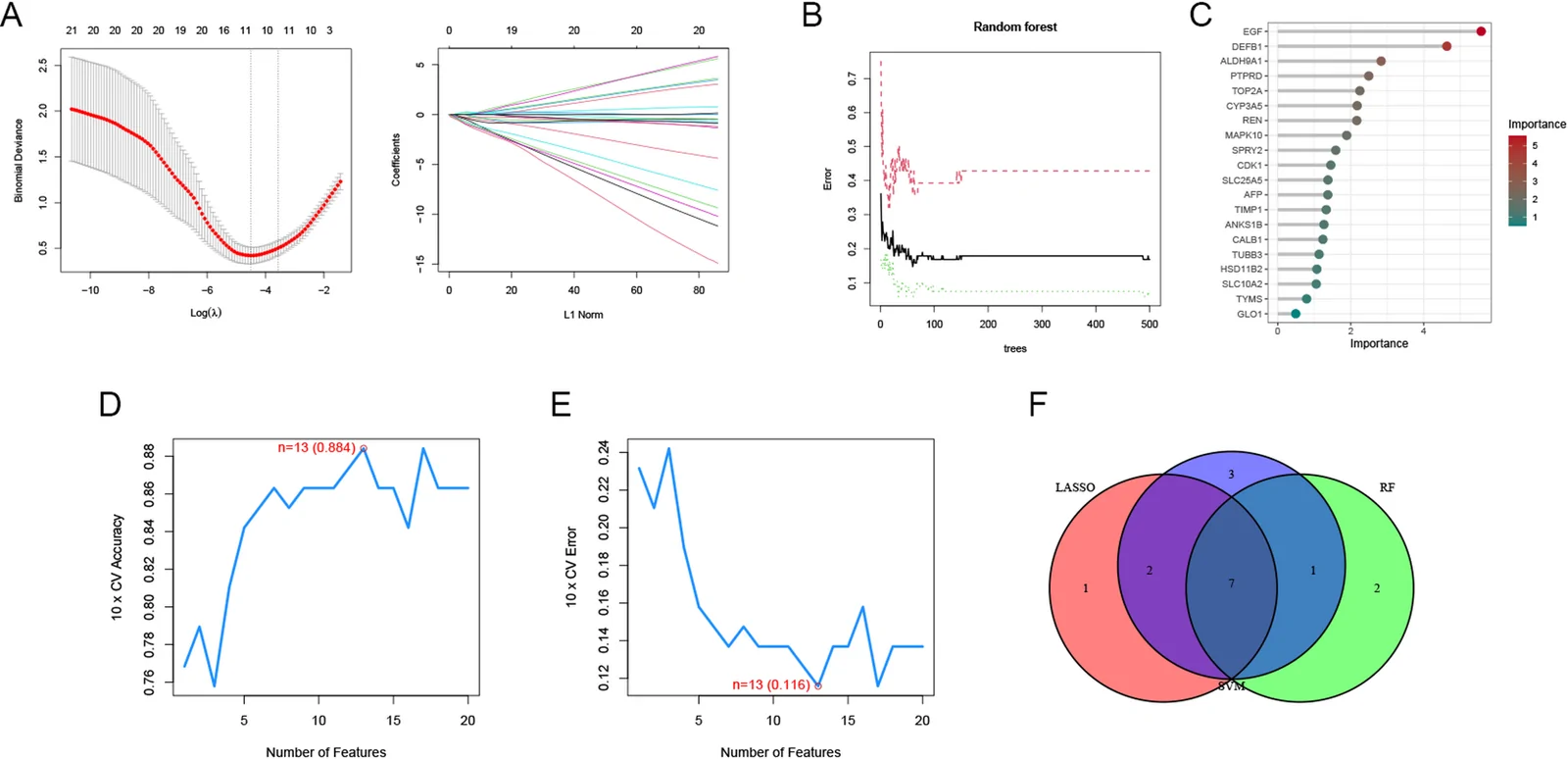
Identification of diagnostic CRRGs in saAKI patients. (**A**) LASSO regression analysis of 21 candidate CRRGs. (**B**–**C**) Identification of characteristic importance based on random forest. (**D**-**E**) The SVM-RFE algorithm was used to screen genes. (**F**) The overlapping CRRGs identified via the Lasso, random forest and SVM-RFE algorithms were defined as diagnostic CRRGs

On the basis of the candidate CRRGs, we used the Lasso, RF and SVM-RFE algorithms to further screen diagnostic CRRGs. With the Lasso algorithm, we obtained 10 CRRGs after removing redundant variables (Fig. 3A). Using the RF algorithm, we selected the top 10 genes on the basis of their importance (Fig. 3B–C). With the SVM-RFE algorithm, when the number of eigengenes was 13, the accuracy was the highest (Fig. 3D–E). By intersecting the 3 machine learning results, we determined 7 diagnostic CRRGs (MAPK10, REN, CDK1, DEFB1, SPRY2, EGF and PTPRD) (Fig. 3F).

### Construction and evaluation of a diagnostic nomogram

**Fig. 4:**
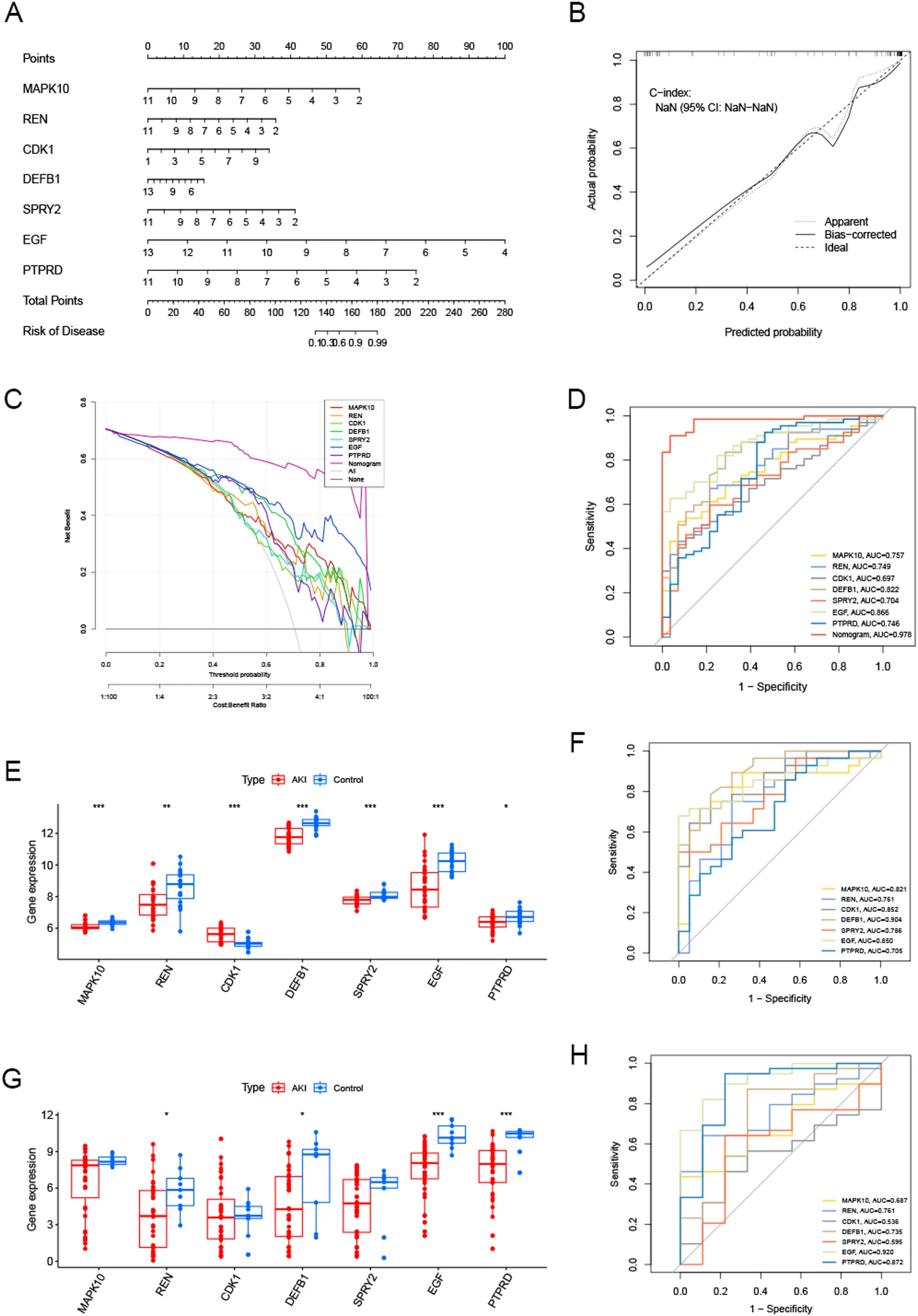
Construction and evaluation of a diagnostic nomogram for saAKI. (**A**) Nomogram based on 7 diagnostic CRRGs. (**B**) The calibration curve shows that the predicted results are very close to the actual results. (**C**) The DCA results show that this nomogram has the greatest net benefit in predicting prognosis. (**D**) The ROC curve shows that the nomogram has the highest AUC value. (**E**) Expression levels of 7 diagnostic CRRGs in the GSE30718 dataset. (**F**) ROC curves were used to evaluate the diagnostic performance of the 7 CRRGs in the GSE30718 dataset. (**G**) Expression levels of 7 diagnostic CRRGs in the GSE139061 dataset. (**H**) ROC curves were used to evaluate the diagnostic performance of the 7 CRRGs in the GSE139061 dataset. *P < 0.05, **P < 0.01, ***P < 0.001

To further elucidate the role of CRRGs in the early diagnosis of saAKI, we constructed a diagnostic nomogram based on 7 diagnostic CRRGs (Fig. 4A). The calibration curve revealed that the predicted data of the model were in good agreement with the actual results (Fig. 4B). The DCA results revealed that the patient outcomes evaluated by this model had a greater net benefit than those evaluated by the CRRGs did (Fig. 4C). The diagnostic ROC curve revealed that the model had a much greater AUC value than the CRRGs did, reaching 0.979 (Fig. 4D). Among the seven genes, DEFB1 and EGF had relatively high AUC values of 0.822 and 0.866, respectively. These evaluations revealed that our nomogram had high diagnostic efficacy. The GSE255281 dataset was used as an external validation cohort to evaluate the predictive efficacy of the nomogram. Interestingly, the calibration curve revealed that the predicted result was very close to the actual result (Supplementary Fig. 1A), and the AUC value was close to 1.000 (Supplementary Fig. 1B). These results also indicate that the model has high accuracy, but a larger sample cohort is still needed for further evaluation. To measure the diagnostic power of the 7 CRRGs in depth, we performed difference and ROC curve analyses in 2 GEO datasets. Interestingly, we found that the expression of 4 CRRGs (REN, DEFB1, EGF, and PTPRD) in saAKI was significantly downregulated in 2 datasets, which was consistent with the results in the combined dataset (Fig. 4E, G). The area under the curve (AUC) values of these 4 CRRGs for the diagnosis of saAKI were greater than 0.700 (Fig. 4F, H). Therefore, we used these 4 CRRGs as hub genes for saAKI and included them in subsequent studies.

### Assessment of immune cell infiltration

**Fig. 5:**
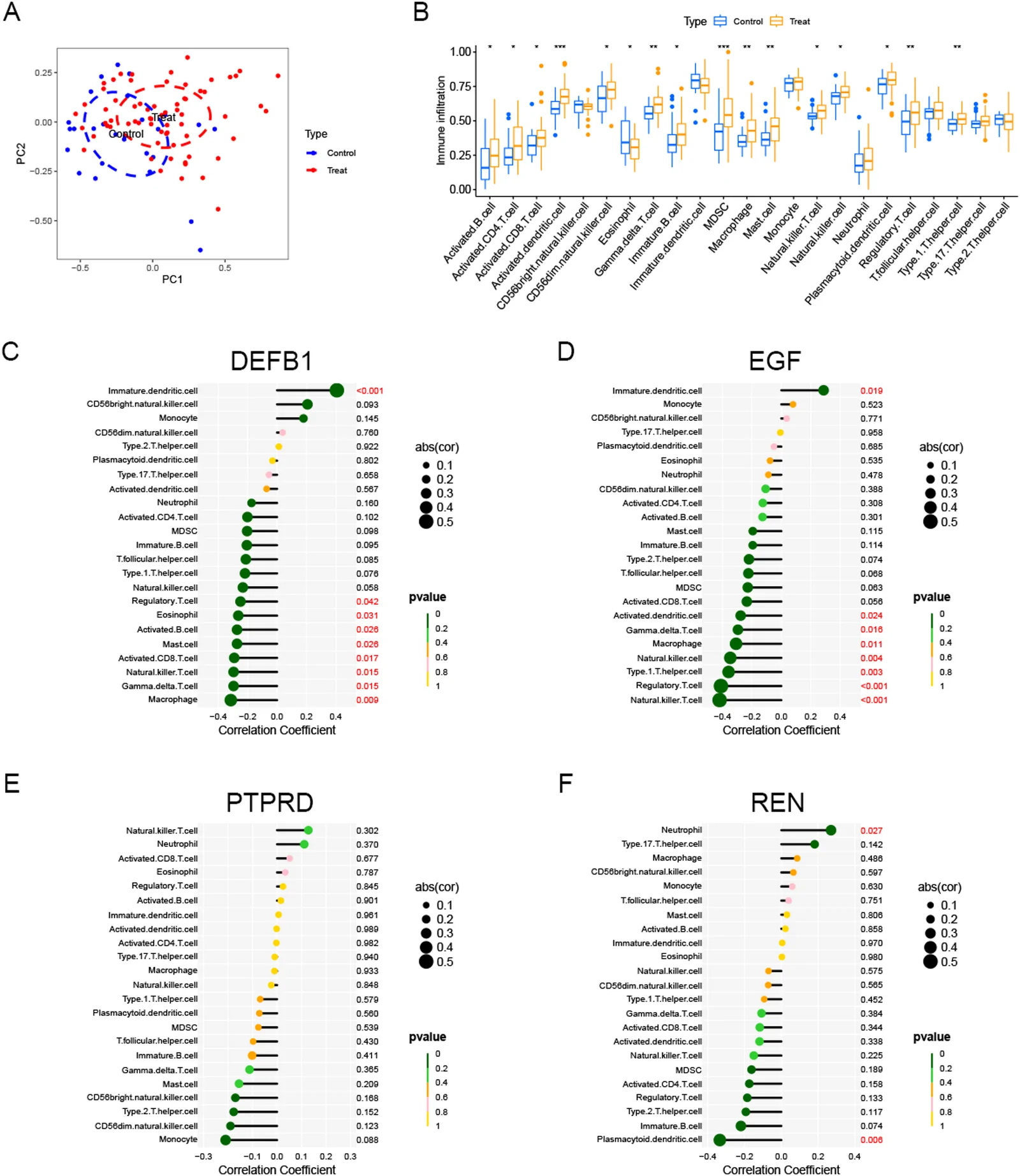
SsGSEA was used to analyze the level of immune infiltration in saAKI. (**A**) PCA results revealed a difference in the level of immune cell infiltration between the saAKI and control samples. (**B**) Infiltration levels of 23 immune cells in the saAKI and control samples. (**C**–**F**) Correlations between the expression of the 4 hub CRRGs and immune cell infiltration. *P < 0.05, **P < 0.01, ***P < 0.001

The infiltration level of immune cells in saAKI samples was evaluated utilizing the ssGSEA algorithm. The PCA results revealed a difference in the level of immune cell infiltration between the saAKI and control samples (Fig. 5A). Compared with those in the control sample, the infiltration of 15 types of immune cells, namely, activated B cells, activated CD4 + T cells, activated CD8 + T cells, activated dendritic cells, CD56-low natural killer cells, gamma delta T cells, immature B cells, MDSCs, macrophages, mast cells, natural killer T cells, natural killer cells, plasmacytoid dendritic cells, regulatory T cells, and type 1 T helper cells, increased, whereas the infiltration of eosinophils decreased (Fig. 5B). In addition, the correlation between immune cells and hub CRRGs was further evaluated. DEFB1 was positively correlated with immature dendritic cells and negatively correlated with macrophages, gamma delta T cells, natural killer T cells, activated CD8 T cells, mast cells, activated B cells, eosinophils and regulatory T cells (Fig. 5C). EGF expression was positively correlated with immature dendritic cells and negatively correlated with natural killer T cells, regulatory T cells, type 1 T helper cells, natural killer cells, macrophages, gamma delta T cells and activated dendritic cells (Fig. 5D). REN was positively correlated with the level of neutrophils and negatively correlated with the level of plasmacytoid dendritic cells (Fig. 5F). However, PTPRD was not associated with immune cell infiltration (Fig. 5E). These data suggest immune dysregulation in saAKI and that the expression of CRRGs may be involved in the regulation of immune processes in saAKI.

### GSVA and GSEA of hub CRRGs in saAKI

**Fig. 6:**
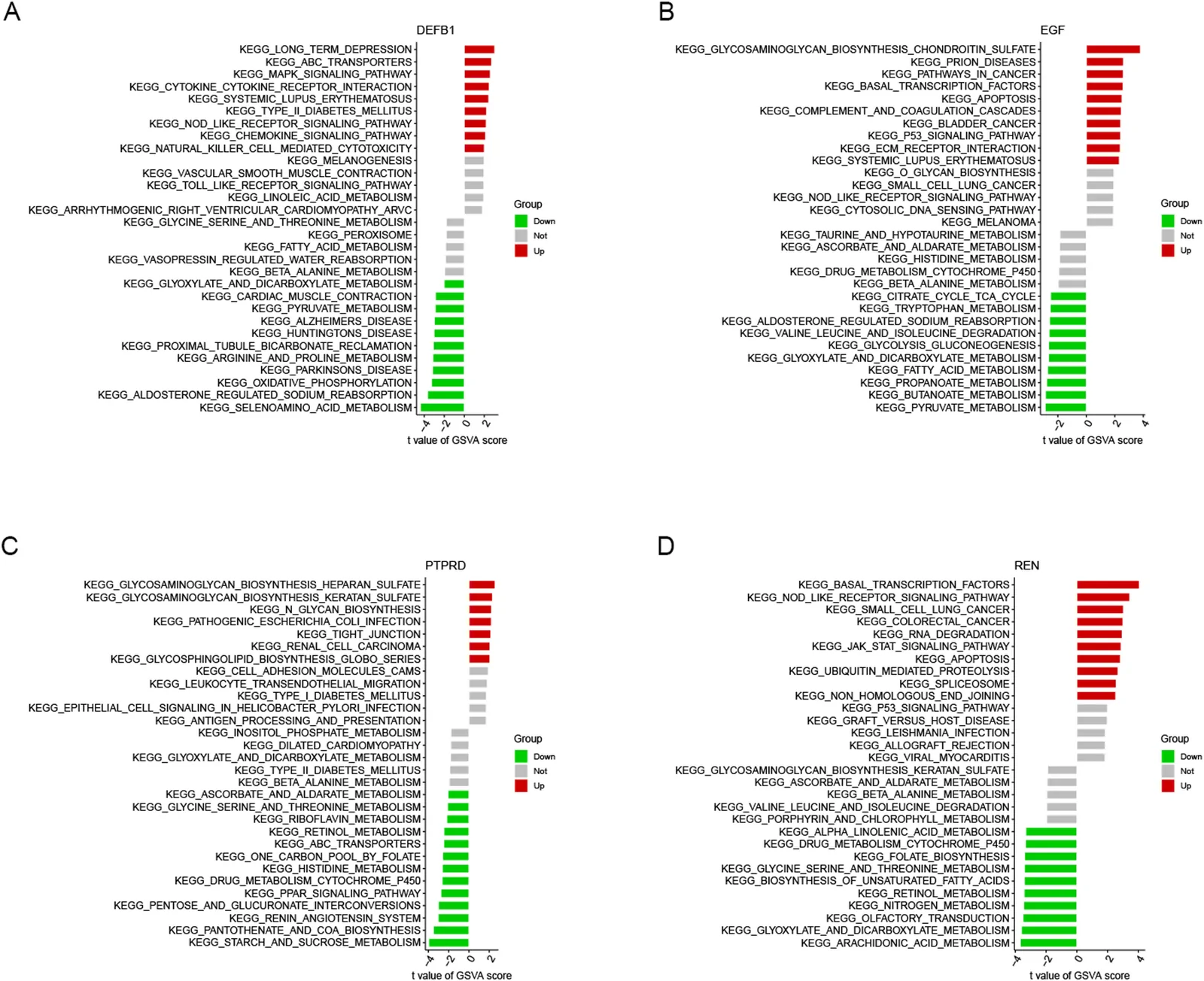
GSVA based on hub CRRG expression levels. The KEGG gene set was used for GSVA to evaluate pathways related to (**A**) DEFB1, (**B**) EGF, (**C**) PTPRD and (**D**) REN in saAKI

**Fig. 7:**
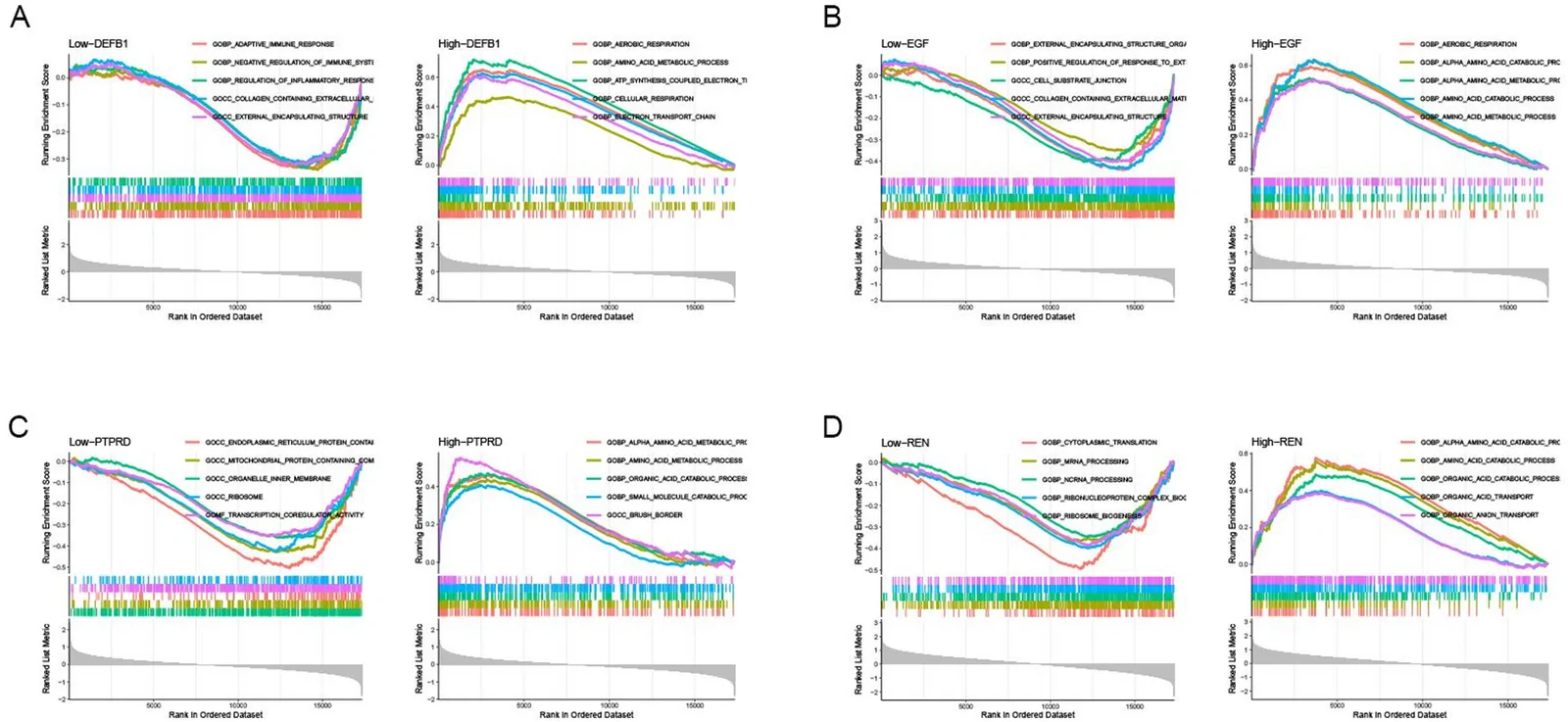
GSEA based on hub CRRG expression levels. (**A**) GO analysis via GSEA in the low- and high-DEFB1 groups. (**B**) GO analysis via GSEA in the low- and high-EGF groups. (**C**) GO analysis via GSEA in the low- and high-PTPRD groups. (**D**) GO analysis via GSEA in the low- and high-REN groups

The KEGG gene set was used for GSVA to evaluate hub CRRG-related pathways in saAKI. Natural killer cell-mediated cytotoxicity, chemokine signaling, NOD-like receptor signaling, cytokine–cytokine receptor interactions, and MAPK signaling pathways were significantly enriched in the high DEFB1 expression group (Fig. 6A). In contrast, renal function and metabolism-related pathways, such as selenoamino acid metabolism, aldosterone-regulated sodium reabsorption, oxidative phosphorylation, arginine and proline metabolism, proximal tubule bicarbonate recovery, pyruvate metabolism, and glyoxylate and dicarboxylate metabolism, were enriched in the low DEFB1 expression group (Fig. 6A). For EGF, ECM receptor interaction, p53 signaling, complement and coagulation cascades, apoptosis, and basal transcription factor pathways were highly enriched in the high-expression group (Fig. 6B). In the low-EGF group, we observed enrichment of many metabolism-related pathways (Fig. 6B). We found that the high expression of PTPRD was closely linked to glycosphingolipid biosynthesis globo series, tight junctions, pathogenic *Escherichia coli* infection, and N-glycan biosynthesis (Fig. 6C). Interestingly, enrichment of many metabolism-related pathways was also observed in the low-PTPRD group (Fig. 6C). With respect to REN, we found that apoptosis, JAK STAT signaling, RNA degradation, NOD LIKE receptor signaling, and basic transcription factor pathways were enriched in the high-expression group, whereas metabolism-related pathways were enriched mainly in the low-REN group (Fig. 6D). The GO gene set was used for GSEA enrichment analysis. We found that many biological functions related to immunity, inflammation, metabolism and material transport were enriched in different subgroups of these 4 CRRGs (Fig. 7).

**Fig. 8:**
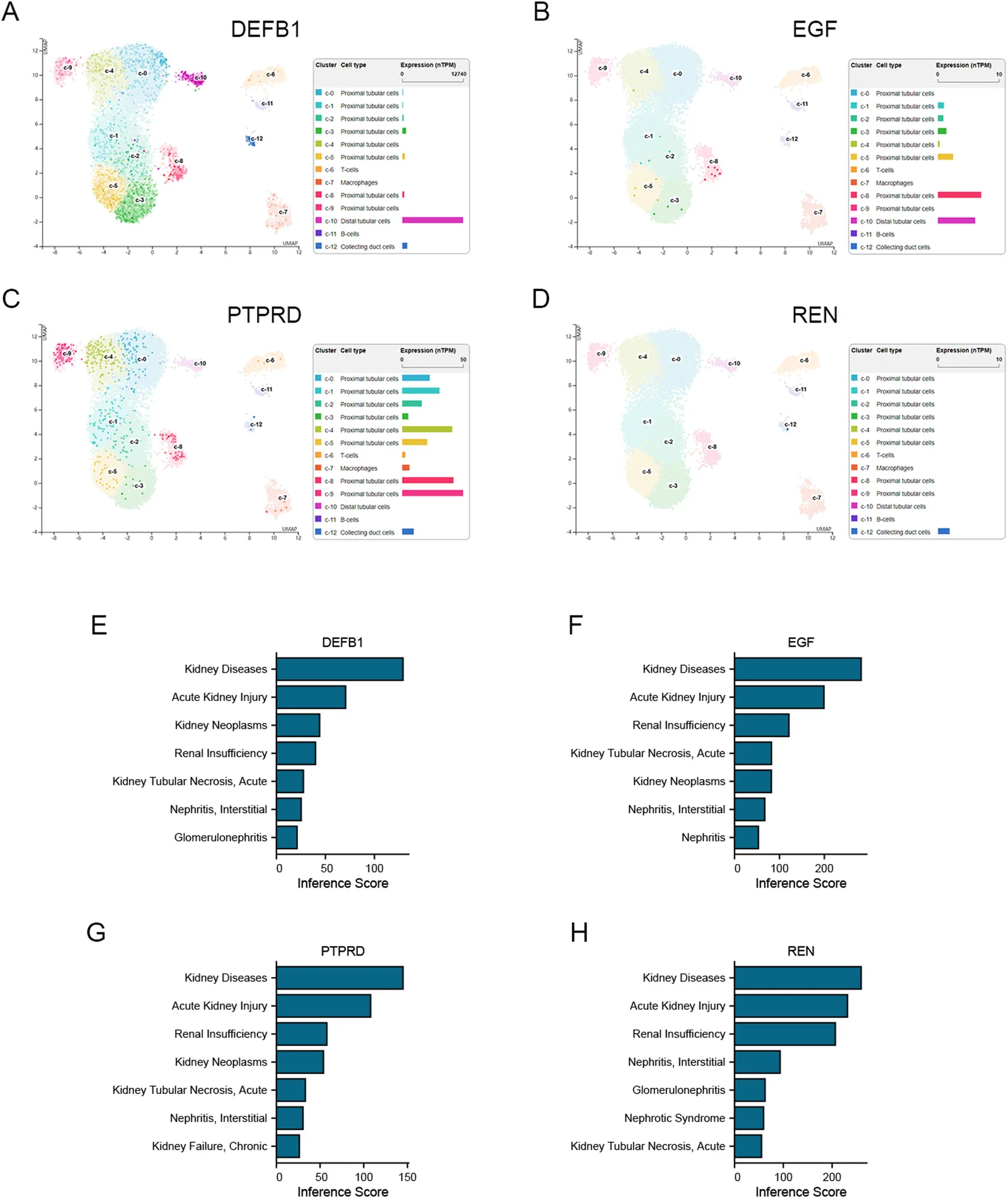
Correlation of hub CRRGs with kidney disease. (**A**–**D**) Using single-cell data from the HPA database, the localization of DEFB1, EGF, PTPRD and REN in the kidney was investigated. The color represents the type of cells. (**E**–**H**) Correlations between the expression of DEFB1, EGF, PTPRD and REN and inferred scores in kidney disease

Using single-cell data from the HPA database, the localization of hub CRRGs in the kidney was investigated. The results revealed that DEFB1 was enriched mainly in distal tubular cells (Fig. 8A). High levels of EGF and PTPRD were detected in distal tubular cells and proximal tubular cells (Fig. 8B–C). REN was expressed mainly in collecting duct cells (Fig. 8D). Finally, the CTD data confirmed that 4 hub genes might be involved in the occurrence and development of AKI or other kidney diseases (Fig. 8E-H).

### Validation of DEFB1, EGF, REN and PTPRD expression

**Fig. 9:**
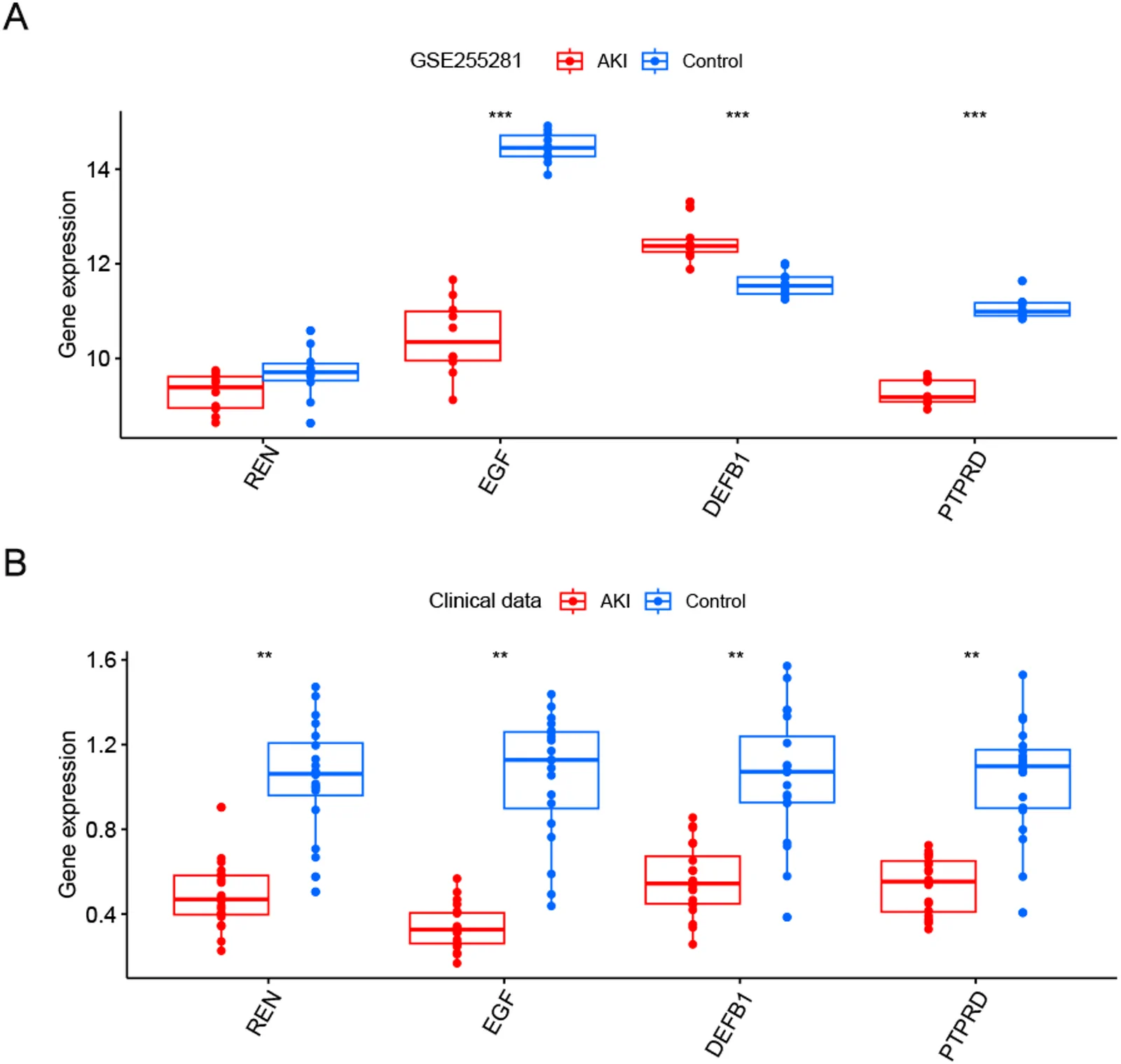
Verification of the expression of DEFB1, EGF, REN and PTPRD in (**A**) the GSE255281 dataset and (**B**) our clinical samples. **p < 0.01, ***p < 0.001

In the GSE255281 dataset, the expression of REN, EGF and PTPRD showed the same trend in saAKI, but only that of EGF and PTPRD was significantly downregulated (Fig. 9A). RT‒qPCR analysis revealed that the expression of DEFB1, EGF, REN and PTPRD was lower in patients with saAKI than in controls (Fig. 9B). These verification results suggest that these CRRGs might be involved in the pathogenesis of saAKI.

## Discussion

Sepsis is among the most common causes of AKI [29]. The occurrence and mechanism of the development of saAKI are complex, and inflammatory immune disorders are considered to be among the key mechanisms [30]. The production of several inflammatory chemokines and reactive oxygen species by the kidney following the occurrence of sepsis further damages various organs of the body and increases the risk of mortality [3]. Recent discoveries have further elucidated the complex mechanisms involved in the development of AKI. Zhong et al. demonstrated that the inflammatory factor LIGHT aggravates inflammation and promotes kidney injury through the TLR4-Myd88-NF-κB signaling pathway [31]. Wang et al. reported the protective role of PINK1-PARK2-OPTN-mediated mitophagy in saAKI [32]. The existing findings indicate a close relationship between circadian rhythm and AKI, but its exact role in AKI remains unclear. Therefore, in-depth study of the biological role of CRRGs is beneficial for improving the diagnosis and treatment of AKI. In this study, we integrated the Lasso, SVM and RF algorithms to identify 7 diagnostic CRRGs in saAKI (MAPK10, REN, CDK1, DEFB1, SPRY2, EGF and PTPRD). Moreover, we constructed a predictive nomogram based on these 7 diagnostic CRRGs, which showed strong diagnostic efficacy. Through the evaluation of 2 subdatasets, we ultimately identified only 4 CRRGs as hub genes for saAKI (DEFB1, EGF, REN and PTPRD). Furthermore, RT‒qPCR revealed that DEFB1, EGF, REN and PTPRD expression was lower in patients with SaAKI than in healthy controls. In the GSE255281 dataset, the expression of REN, EGF and PTPRD showed the same trend in saAKI, but only that of EGF and PTPRD was significantly downregulated. These verification results suggest that these CRRGs might be involved in the pathogenesis of saAKI.

Immune and inflammatory activation affects the occurrence and development of AKI, and the immune system is significantly influenced and regulated by circadian mechanisms [33–35]. Unbalanced dendritic cells lead to abnormal immune homeostasis and are associated with abnormal inflammatory activation [36]. The immunoinflammatory process mediated by regulatory T cells may be related to their promotion of fibrogenesis and cytokine production [37, 38]. Natural killer T cells may form proinflammatory or anti-inflammatory microenvironments with different characteristics depending on the type of antigen-presenting cell [39]. In this study, the expression of DEFB1 and EGF was positively correlated with immature dendritic cells and negatively correlated with natural killer T cells, gamma delta T cells and regulatory T cells. DEFB1 is an important component of antimicrobial peptides in various epithelial tissues and has been shown to be associated with a variety of infectious diseases [40, 41]. DEFB1 is downregulated in chronic kidney disease tissues, and activation of DEFB1 can significantly reduce inflammation; thus, DEFB1 may be an effective therapeutic target for chronic kidney disease [42]. In our study, DEFB1 was downregulated in saAKI samples, and we speculated that when inflammation occurs in the body, DEFB1 expression in the kidney is reduced, thereby causing or aggravating kidney injury. Previous studies have shown that EGF is decreased in the tissue and urine of AKI patients and may serve as a biomarker for AKI [43, 44]. Zeid et al. reported that EGF deficiency accelerates the progression of renal fibrosis and is associated with activation of the β-catenin/mTOR pathway [45]. EGF regulates the expression of antiapoptotic proteins through the activation of the PI3K/AKT, RAS/ERK and JAK/STAT pathways and can effectively block the apoptotic pathway [46]. In our study, EGF expression was significantly downregulated in AKI, and GSVA revealed that it was associated with the SA-AKI apoptosis pathway, which was similar to previous conclusions. PTPRD variants affect nonalcoholic fatty liver disease by promoting steatosis, inflammation and fibrosis [47]. Mutations in the REN gene are known to cause renin abnormalities in the body and have been associated with a variety of kidney diseases, including autosomal recessive renal tubular dysgenesis, autosomal dominant tubulointerstitial kidney disease and chronic kidney disease [48–50]. Interestingly, this study revealed that both PTPRD and REN were significantly downregulated in patients with saAKI, but their role in patients with saAKI needs to be further explored.

Through GSEA and GSVA, we revealed that CRRGs may be involved in the regulation of many metabolism-related functions and pathways. The circadian system has been shown to play a universal role in regulating metabolism [51, 52]. On the other hand, metabolic dysfunction is a critical contributor to the pathogenesis of AKI, and correcting metabolic abnormalities is a promising strategy for reversing or preventing AKI [53, 54]. Therefore, we believe that abnormal CRRG expression may disrupt renal metabolic balance, thus exacerbating the development of saAKI. Our study may help to elucidate the relationships among circadian rhythm, metabolism and AKI.

To our knowledge, only a few studies have used sophisticated machine learning algorithms to identify biomarkers in saAKI; thus, we believe these findings are useful addition to the existing saAKI biomarkers. Moreover, our data may provide new ideas for further research into the immune and metabolic mechanisms underlying the relationship between the circadian rhythm and saAKI. However, some limitations of this study need to be considered. At present, public datasets of saAKI are relatively scarce. Although the internal and external validation results indicate that our model has high accuracy, the sample size of the current dataset is relatively small, and a larger and more diverse cohort is still needed to verify the accuracy of this diagnostic nomogram model. Second, the clinical samples in this study were relatively small. The lack of sufficient clinical blood, urine or tissue samples for verification is another limitation of this study. In future studies, we will evaluate the diagnostic efficacy of identified CRRGs in a clinical cohort and conduct longitudinal studies to track changes in the expression of hub CRRGs and their impact on saAKI outcomes. Finally, although this study initially indicates that CRRGs may affect the immune and metabolic pathways of patients with saAKI, this merely provides an idea. Further in vivo and in vitro experiments are still needed to determine the specific mechanism involved.

## Conclusions

In summary, this research utilized comprehensive machine learning methods to construct a CRRG-based diagnostic model for saAKI. The identified hub CRRGs may be potential biomarkers of saAKI, and this study may provide new strategies for the diagnosis and treatment of saAKI.

## Supplementary Information

Supplementary Material 1: Supplementary Table 1. Sequences of the primers designed for RT-qPCR.

Supplementary Material 2: Supplementary Table 2. The clinical characteristics of the saAKI patients.

Supplementary Material 3: Supplementary Figure 1. (A) The calibration curve for external verification group; (B) The ROC curve for the external validation group.

## Data Availability

The data presented in this study are available on request from the corresponding author.

## Abbreviations

AKI: Acute kidney injury
saAKI: Sepsis-associated acute kidney injury
CRRG: Circadian rhythm-related gene
DEG: Differentially expressed gene
GEO: Gene expression omnibus
Kim-1: Kidney injury molecule-1
Lasso: Least absolute shrinkage and selector operation
MAD: Median absolute deviation
NGAL: Neutrophil gelatinase-associated lipocalin
PPI: Protein-protein interaction
PCA: Principal component analysis
RF: Random forest
RAAS: Renin-angiotensin-aldosterone
SVM-RFE: Support vector machine recursive feature elimination
WGCNA: Weighted gene co-expression network analysis
