# Supplementary material for: Comprehensive molecular analyses of a circadian rhythm-related gene diagnostic model and immune infiltration using machine learning methods in sepsis-associated acute kidney injury
Source: Hereditas. 2026 Jun 11;163:88. doi: 10.1186/s41065-026-00698-3 (PMC13491666; doi:10.1186/s41065-026-00698-3)
Supplement: Supplementary file 1 [file 41065_2026_698_MOESM1_ESM.docx]

**Supplementary table 1 Sequences of the primers designed for RT-qPCR.** se

| Gene | Forward sequence | Reverse sequence |
| --- | --- | --- |
| DEFB1 | CCTCAGGTGGTAACTTTCTC | CACTTCTGCGTCATTTCTTCT |
| EGF | TTCCTCTTAGCCCAGTATCC | GGCATAAACCATTCCCATCTG |
| REN | AGGCTGTTTGATTATGTCGTG | GGTGAGCGTGTATTCTTTGC |
| PTPRD | GTGCCAGATTACTCAGTGCT | AGTGCTCCATCGGATTCTAC |
| ACTB | GTGACAGCAGTCGGTTGGAG | GTGGGGTGGCTTTTAGGATG |
