# Supplementary material for: Comprehensive molecular analyses of a circadian rhythm-related gene diagnostic model and immune infiltration using machine learning methods in sepsis-associated acute kidney injury
Source: Hereditas. 2026 Jun 11;163:88. doi: 10.1186/s41065-026-00698-3 (PMC13491666; doi:10.1186/s41065-026-00698-3)
Supplement: Supplementary file 2 [file 41065_2026_698_MOESM2_ESM.docx]

| **ID**  **Supplementary Table 2 The clinical characteristics of the saAKI patients.** | **Consciousness** | **GENDER** | **Age (years)** | **Pulse(beats/minute)** | MAP(mmHg) | **Scr(μmol/L)** | **24-hour urine volume(ml)** | **Pa/FiO2** | **Lac** | **PLT** | **Bilirubin(umol/L)** | **SOFA score** |
| --- | --- | --- | --- | --- | --- | --- | --- | --- | --- | --- | --- | --- |
| 1 | normal | M | 83 | 97 | 68.33 | 231 | 1500 | 494 | 4.44 | 112 | 20.9 | 4 |
| 2 | normal | M | 67 | 104 | 78.00 | 132 | 1250 | 290 | 3.8 | 169 | 35.5 | 5 |
| 3 | normal | M | 53 | 96 | 92.00 | 191 | 1800 | 330 | 1.14 | 342 | 9.6 | 3 |
| 4 | normal | M | 51 | 117 | 67.00 | 103 | 2650 | 168 | 4.09 | 448 | 32.2 | 6 |
| 5 | normal | M | 55 | 92 | 77.00 | 94 | 1950 | 308 | 1.2 | 169 | 21.9 | 2 |
| 6 | normal | M | 50 | 98 | 73.33 | 417 | 800 | 370 | 2.93 | 560 | 8.2 | 4 |
| 7 | normal | M | 42 | 99 | 104.00 | 1474 | 2800 | 436 | 1.8 | 547 | 17.2 | 4 |
| 8 | normal | M | 59 | 126 | 86.33 | 201 | 3860 | 299 | 1.46 | 432 | 6.4 | 4 |
| 9 | normal | M | 52 | 103 | 81.33 | 150 | 1500 | 310 | 2.4 | 209 | 51.7 | 4 |
| 10 | normal | F | 57 | 103 | 76.00 | 230 | 3550 | 388 | 1.4 | 176 | 8 | 3 |
| 11 | normal | M | 75 | 123 | 74.33 | 154 | 1250 | 169 | 7.8 | 254 | 50.2 | 6 |
| 12 | normal | F | 59 | 117 | 68.33 | 445 | 290 | 112 | 16.2 | 24 | 24.5 | 12 |
| 13 | normal | M | 72 | 118 | 76.67 | 262 | 2070 | 272 | 7.4 | 387 | 11.6 | 4 |
| 14 | normal | M | 68 | 126 | 90.67 | 341 | 610 | 203 | 2.2 | 304 | 12.7 | 5 |
| 15 | normal | M | 71 | 121 | 75.33 | 133 | 2500 | 286 | 7.9 | 35 | 31.5 | 7 |
| 16 | normal | F | 36 | 120 | 102.67 | 719 | 330 | 315 | 1.1 | 260 | 156 | 8 |
| 17 | normal | F | 66 | 92 | 43.67 | 346 | 1500 | 433 | 5.3 | 76 | 164.5 | 9 |
| 18 | normal | M | 68 | 112 | 69.67 | 368 | 320 | 200 | 3.9 | 56 | 428 | 12 |
| 19 | normal | M | 53 | 106 | 103.67 | 247 | 2600 | 350 | 1 | 411 | 4.7 | 3 |
| 20 | normal | M | 52 | 112 | 66.33 | 162 | 1860 | 481 | 5.6 | 731 | 5.6 | 2 |
